# ﻿Mammal diversity and tourism influence in an under-investigated region of Costa Rica

**DOI:** 10.3897/zookeys.1260.128800

**Published:** 2025-11-14

**Authors:** Federica Fonda, Liam Vezzani, Luis Ángel Mena Aguilar, Carlos Andrés Venegas-Elizondo, Alexander Bolaños Brenes, Dayron Manuel Lopez, Giuseppe Romeo, Dario Sonetti, Matteo Dal Zotto

**Affiliations:** 1 Department of Life Science, University of Trieste, Via L. Giorgieri 10, 34127 Trieste, Italy University of Trieste Trieste Italy; 2 Department of Life Sciences, University of Modena and Reggio Emilia, Via Campi 213/d, 41125 Modena, Italy University of Modena and Reggio Emilia Modena Italy; 3 PBL Netherlands Environmental Assessment Agency, Bezuidenhoutseweg 30, 2594 AV The Hague, Netherlands PBL Netherlands Environmental Assessment Agency The Hague Netherlands; 4 Asociación Ecológica Paquera, Lepanto y Cóbano, PO Box 25-5353, Jicaral, Puntarenas, Costa Rica Asociación Ecológica Paquera, Lepanto y Cóbano Jicaral Costa Rica; 5 Associazione Foreste per Sempre OdV, Via D’Avia Sud 65/a, 41126 Modena, Italy Associazione Foreste per Sempre OdV Modena Italy

**Keywords:** Activity patterns, camera trapping, checklist, conservation, mammal community, temporal overlap

## Abstract

Costa Rica is one of the most biodiverse countries in the world, hosting almost 4% of all known mammals. Although the local mammals have undoubtedly been more investigated than those of other Central American countries, some regions still appear poorly known to date, such as the Nicoya Peninsula, NW Costa Rica. Our research focuses on the Karen Mogensen Wildlife Refuge, an important component of the Nicoya Peninsula Biological Corridor. This investigation represents the first comprehensive study of mammals of this area, with the aims to provide baseline information on the mammal community and tourism management. The investigation during a 6-year period (2018–2023) revealed the presence of 60 species, belonging to 50 genera, 23 families, and 8 orders, indicating a high species richness of the area. Among these, we highlight the occurrence of the globally Vulnerable *Alouatta palliata* and *Cebus imitator* and the Near Threatened *Leopardus wiedii* and *Vampyrum spectrum*, along with the recently recognised *Lontra annectens*, whose status is still Not Evaluated. The influence of tourism on activity patterns of mammals was also investigated. Using camera trapping, 13 species were detected in 537 trap-days and divided based on functional traits. No evidence of impacts caused by the presence of tourists was found on these species. This suggests that the current conservation policies of the Wildlife Refuge appear to be incorporating ecotourism in a wise and thoughtful manner. The information gathered will be a useful tool for the development and implementation of conservation strategies and actions at the local scale in the near future.

## ﻿Introduction

The Central American Costa Rica, one of the 36 world biodiversity hotspots (Mittermeier et al. 2005), represents only 0.03% of the world’s land surface (Rodríguez and Chinchilla 1996; Reid 2009) but hosts almost 4% of all mammals’ species in the World (Wainwright 2007; Reid and Gόmez Zamora 2022; Mammal Diversity Database 2025). Several factors contribute to this diversity, from the tropical climate, which provides conditions that allow the growth of many autotrophic and heterotrophic organisms, to the four mountain ranges as well as the weather systems blowing in from both oceans, creating a dense and varied mosaic of habitats (Huettmann 2015). Costa Rica’s diversity is also influenced by its very position right between North and South America, connected to each other on account of the formation of the Panamanian land bridge approximately 2.8 million years ago (Woodburne 2010).

Today, the mammal conservation in Costa Rica is very delicate on account of two fundamental aspects. On the one hand, the second half of the last century was a dismal period for many Costa Rican mammals as a consequence of habitat loss caused by deforestation and pollution coming from the industrial, domestic, and agricultural sectors (Evans 1999). On the other hand, in 1970 a national park system was created and 28% of the country, in one form or another, was brought under its protection (Evans 1999). Consequently, ecotourism has grown enormously during the last decade and for many landowners, protecting forests and opening them up to tourism has become the most lucrative option. Several examples, from Costa Rica (Fletcher 2012; Hunt et al. 2015; Lopez Gutierrez et al. 2020) to many other regions of the globe (Tisdell and Wilson 2002; McConnell and Kull 2014; Buckley et al. 2016), show that ecotourism provides socio-economic and environmental sustainability.

Species coexistence is ruled by several and complex mechanisms (Chesson 2000) and is based on the partitioning of ecological niches (Amarasekare 2003). Despite being a complex concept to define, a niche is generally described by three dimensions: temporal, resource, and spatial (Case and Gilpin 1974; Albrecht and Gotelli 2001; Amarasekare 2003). Species that coexist in an area must differ in at least one of these dimensions (Gause 1934). Despite being regarded as the least important, the temporal dimension often plays a key role in species’ behaviours and interactions, and their associated consequences for community structure (Monterroso et al. 2014; Frey et al. 2017). There is a strong dependence between the species diel activity patterns and their internal “clocks” (Aschoff 1966) that is species-, sex-, and age-dependent, but several factors can affect this “clock” with consequent changes in phenotypic plasticity (Monterroso et al. 2014; Martín-Díaz et al. 2018). How species use time and distribute their activity within the diel cycle is an important aspect of animal ecology and behaviour that provides valuable information about their ecological niche (Schoener 1974; Frey et al. 2017). At the community level, understanding how species partition time contribute to explain the mechanisms facilitating stable coexistence (Carothers and Jaksić 1984; Kronfeld-Schor and Dayan 2003; Frey et al. 2017). Indeed, patterns of daily activity of the species determine the biotic (e.g., predation, competition), abiotic (e.g., light level, temperature), and anthropogenic factors to which they are exposed (Schoener 1974; Hut et al. 2012; Cox et al. 2021). For example, predators adapt their activity to maximise the probability of prey encounter and, contrarily, preys reduce their activity when predators are active (Foster et al. 2013; Monterroso et al. 2013). In addition, species with similar ecology and morphology minimise interspecific competition through temporal segregation (Lucherini et al. 2009; Di Bitetti et al. 2010; Monterroso et al. 2014).

The presence of humans in wilderness and protected areas can disturb wildlife, inducing behavioural changes and shifts in diel activity patterns, even in response to non-lethal human activities such as hiking and recreational trekking (Taylor and Knight 2003). For example, in the absence of tourists, leopards (*Panthera pardus*) exhibit increased diurnal activity compared to periods when tourists are present (Ngoprasert et al. 2017). Similarly, other large carnivores such as jaguars (*Panthera onca*) and pumas (*Puma concolor*) have shown increased nocturnality in response to human presence (Ouboter et al. 2021). This shift to nocturnal behaviour has been extensively documented in a global meta-analysis by Gaynor et al. (2018), which demonstrated that, worldwide, mammals are increasingly becoming nocturnal as a strategy to avoid human disturbance. Mammals of all trophic levels, including carnivores, omnivores, and herbivores, have exhibited increased nocturnality in response to human presence, with diurnal species becoming more nocturnal and crepuscular or nocturnal species further intensifying their nocturnal activity (Gaynor et al. 2018).

Most studies on temporal patterns in species and community ecology have used camera trapping, a method that has proven to be very efficient for this kind of research (Frey et al. 2017). Temporal camera trapping data offer the opportunity to investigate diel activity patterns and temporal partitioning in sympatric mammals (e.g., Di Bitetti et al. 2010; Monterroso et al. 2014; Sunarto et al. 2015), as well as evaluating prey-predator and predator-predator interactions (e.g., Botts et al. 2020; Hernández-Sánchez and Santos-Moreno 2020; Marinho et al. 2020), and effects of human-driven disturbances on wildlife (Wang et al. 2015; Ngoprasert et al. 2017). Camera trapping has also proven to be particularly suitable to assess species richness of an area (Rovero et al. 2014; Rovero and Spitale 2016) and to study rare and elusive animals (Rovero et al. 2010), common characteristics of Neotropical mammals (Thompson 2004; Sanderson and Trolle 2005; Botts et al. 2020).

In this study we investigated the mammal community of the Karen Mogensen Wildlife Refuge, located in the southern part of Nicoya Peninsula, northwestern Costa Rica. This region is one of the most poorly studied in all of Costa Rica (Timm et al. 2009), although it hosts the most threatened forest ecosystem of Central America (Janzen 1988; Huettmann 2015). Indeed, for the Karen Mogensen Wildlife Refuge, as for most of Costa Rican protected areas, ecotourism represents a fundamental income for its maintenance. Our study, which represents the first comprehensive study of the mammals of this protected area, intends to report baseline information on mammal community, species temporal interactions, and human influences. Specifically, we aimed to: (1) create a complete checklist of mammals present in the entire area; (2) examine the diel activity patterns of mammals in the Karen Mogensen Wildlife Refuge; (3) assess the effectiveness of tourism management in the protected area by evaluating variations in the activity rhythm of species and functional groups in relation to the presence or absence of tourists. The documentation of mammalian species richness, along with exploratory analyses aimed at assessing potential impacts of tourism, provides important insights into wildlife behavioural responses to human disturbance within the Wildlife Refuge.

## ﻿Materials and methods

### ﻿Study area

**Figure 1.**
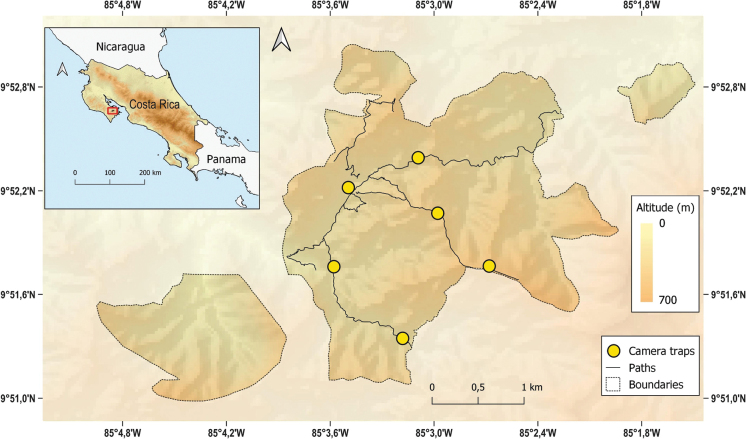
Location of the study area, the Karen Mogensen Wildlife Refuge, northwestern Costa Rica. The areas bordered by dashed lines are parts of the Wildlife Refuge added to the original nucleus over time.

The investigation took place in the Karen Mogensen Wildlife Refuge, SE Nicoya Peninsula, northwestern Costa Rica (Fig. 1; centroid coordinates: 09°51'36"N, 085°03'00"W). This zone is an inland foothill area, and its extension is ca. 1,000 hectares. The altitudes range from 130 to 600 m a.s.l., even though in the vicinities of the Wildlife Refuge altitudes of more than 750 m can be reached (Fig. 1). The whole area is in continuity with surrounding forested environments. The Wildlife Refuge is characterised by the presence of: dry to moist transitional forest, moist forest, dry forest (see the habitat classification by Holdridge 1967), pasture, and grassland. The forested environments consist mainly of second growth and patches of primary forest. For further information see Dal Zotto et al. (2017; 2024) and Lopez et al. (2023). As for the climate, the southernmost part of the Nicoya Peninsula is characterised by a dry season from December to April and a rainy season in the remaining part of the year. Average temperatures throughout the year are over 25 °C, with highs of more than 30 °C in the dry season (https://meteo.unimore.it/meteo/ossgeo/weewx/karen/).

The area underwent significant alterations due to resource exploitation and farming in the past century, mostly cattle pasture. An extraordinary comeback began in 1996, thanks to the joint efforts of local and foreign organisations (Lopez et al. 2023). Currently, the area is included in the Biological Corridor of the Nicoya Peninsula and recognised as a Wildlife Refuge of national relevance.

#### ﻿Data collection

We collected observational data, some of which were obtained from previous surveys based on series of transects resulting in nearly 7 km of linear walk (i.e., Dal Zotto et al. 2017; Lopez et al. 2023), and occasional records by faunistic experts. Most of this information was gained by walking the main paths of the area reported in Fig. 1. As for non-volant mammals, we considered records from direct observations, camera trapping, or unmistakable indirect signs of presence (e.g., carcasses, spraints, faeces, footprints), using the field guides by Wainwright (2007) and Reid and Gómez Zamora (2022), and relying on our experience. No dedicated study was conducted on micromammals, a group that requires specific methods of investigation. For this reason, we reported only few occasional observations of these animals. Regarding bats, in addition to direct observations, over fifteen mist netting sessions, each employing at least five nets, were carried to increase the possibility of identifying additional species. The diagnosis of Chiroptera was based on York et al. (2019). All data were recorded between 2018 and 2023. The habitat types reported are based on a previous investigation led in the study area (Dal Zotto et al. 2017). The systematisation, the scientific and common names of the recorded taxa follow the Mammal Diversity Database (2025).

#### ﻿Camera trapping survey

Camera trapping was used for the six-year investigation period, opportunistically. However, the data for analysing the activity patterns and the influence of tourism were collected in a more structured way from mid-January to mid-April 2019. Data were registered using six camera traps, which worked simultaneously 24 hours a day. Camera traps were placed along the paths in the study area, used also by the tourists, considering the presence of water and animal tracks, at an average distance among them of 753 m (Fig. 1). Each camera trap was placed on a tree at a height of 40–50 cm above ground level and was set to register 10-second videos for each movement detected. Every two weeks they were checked for battery change and data download. All video information, such as the species detected, date, hour, number of individuals, sex, and other relevant notes, were recorded in a dedicated database.

### ﻿Data analyses

#### ﻿Species accumulation curve

The species accumulation curve was used to evaluate the completeness of the species inventory during the camera trapping survey (Ugland et al. 2003; Rovero and Spitale 2016). In general, when the curve reaches an asymptote, all species present in the area have been detected. To assess whether our survey effort was sufficient to detect the majority of species present in the community, we constructed a randomised (1000 times) accumulation curve using the vegan package (Oksanen et al. 2013) in R (R Core Team 2020, v. 4.0.1).

#### ﻿Diel activity pattern of mammals and tourism influence

As almost all the entrances to the Karen Mogensen Wildlife Refuge are controlled by the local association ASEPALECO (Asociación Ecológica Paquera, Lepanto y Cóbano), we were able to identify the days when there was a possibility of human disturbance, i.e., tourists entering the Wildlife Refuge. To assess whether the target species were disturbed by human presence, we tested whether the daily activity patterns of mammals changed between days when tourists were present and days when they were not. After dividing the species detections into two classes, i.e., ‘with tourists’ and ‘without tourists’ days, we conducted exploratory analyses to test whether the presence of tourists influenced the diel activity patterns of: i) the most abundant species (> 40 detections), ii) the entire community, iii) nocturnal and diurnal species, and iv) species grouped by trophic level (herbivore, omnivore, and carnivore; Jones et al. 2016). These analyses aimed to investigate whether species exhibited shift toward increased nocturnality to avoid human disturbance.

To avoid biased results, consecutive records of the species at the same camera trap site were considered independent if there was at least a 1-hour interval between them (Foster et al. 2013).

To assess the selection of each species for different periods of the diel cycle, a time band was assigned to each video depending on the time it was recorded. Following Foster et al. (2013) and Monterroso et al. (2014), four bands were used: Sunset, 1 hour before and after the sunset; Sunrise, 1 hour before and after sunrise; Day, between sunrise and sunset; Night, between sunset and sunrise. Exact sunset and sunrise times were obtained from NOAA (NOAA Global Monitoring Laboratory 2025). Although tropical areas show almost negligible variation in sunset and sunrise times over a short period, an average time was calculated for each month and used to assign the time band. We estimated Ivlev’s Electivity Index (Ivlev 1961), modified by Jacobs (1974), hereafter D, to assess species selection for each time band. Using bootstrap resampling (10,000 replicates; Manly 1997) and recalculating the index for each bootstrap sample, we estimated the average for each time band and species. We classified the species with more than 40 detections as diurnal or mostly diurnal, nocturnal or mostly nocturnal, and cathemeral following the criteria proposed by Gómez et al. (2005). Species detected fewer than 40 times were classified using open databases, such as PanTHERIA (Jones et al. 2016), or the scientific literature (Botts et al. 2020).

Diel activity patterns were estimated non-parametrically through the probability density function using Kernel Density Estimate (Ridout and Linkie 2009) and the Watson’s test (U^2^) was used to assess the uniformity of distribution. To quantify the similarity of the temporal activity pattern of species and groups between the two classes, i.e., ‘with tourists’ and ‘without tourists’ days, we compared the overlap of the probability density functions by the overlap coefficient ∆_i_ (Ridout and Linkie 2009). This coefficient ∆_i_ ranges from 0, no overlap, to 1, complete overlap, and, as suggested by Ridout and Linkie (2009) and Meredith and Ridout (2014), we used ∆_1_ when the sample size was < 75 and ∆_4_ when the sample size was > 75. The precision of the coefficients and 95% confidence intervals were obtained using smoothed bootstrap resampling (10,000 replicates) (Meredith and Ridout 2014; Zimmermann et al. 2016). Therefore, we evaluated activity overlap values following the suggestion of Monterroso et al. (2014): we calculated the percentile of overlap values of the total pairwise comparisons performed. We classified overlap as “low” when ∆ ≤ 50^th^ percentile, “moderate” when 50^th^ percentile < ∆ ≤ 75^th^ percentile, and “high” when ∆ > 75^th^ percentile. To assess whether there were significant differences between the activity patterns for all pairwise combinations, we performed Watson’s two-sample test (two-sample U^2^).

All statistical analyses were performed in R, using circular (Lund and Agostinelli 2017), overlap (Meredith and Ridout 2014), and CircStats (Lund and Agostinelli 2018) packages.

## ﻿Results

### ﻿Species richness

**Figure 2.**
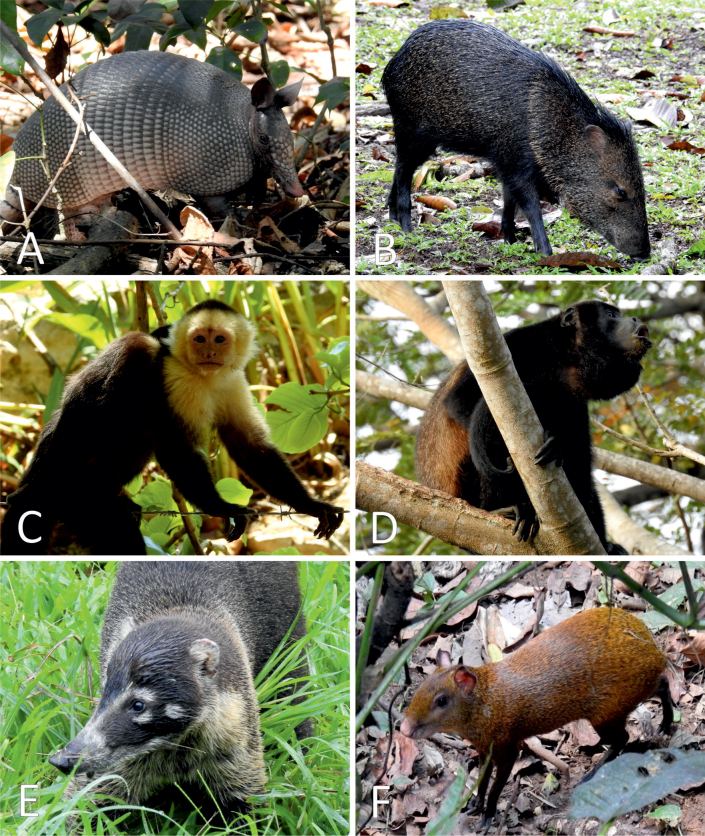
Mammals directly observed at the Karen Mogensen Wildlife Refuge. **A.***Dasypus novemcinctus*; **B.***Dicotyles tajacu*; **C.***Cebus imitator*; **D.***Alouatta palliata*; **E.***Nasua narica*; **F.***Dasyprocta punctata*. Photographs by M. Dal Zotto.

**Figure 3.**
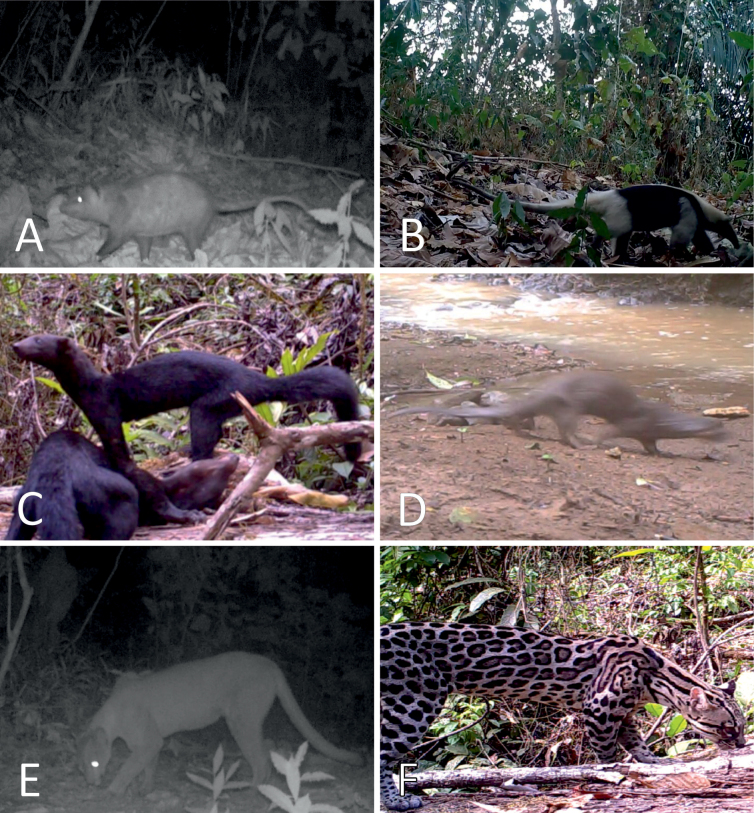
Camera-trapped mammals in the Karen Mogensen Wildlife Refuge. **A.***Didelphis marsupialis*; **B.***Tamandua mexicana*; **C.***Eira barbara*; **D.***Lontra annectens*; **E.***Puma concolor*; **F.***Leopardus pardalis*.

**Figure 4.**
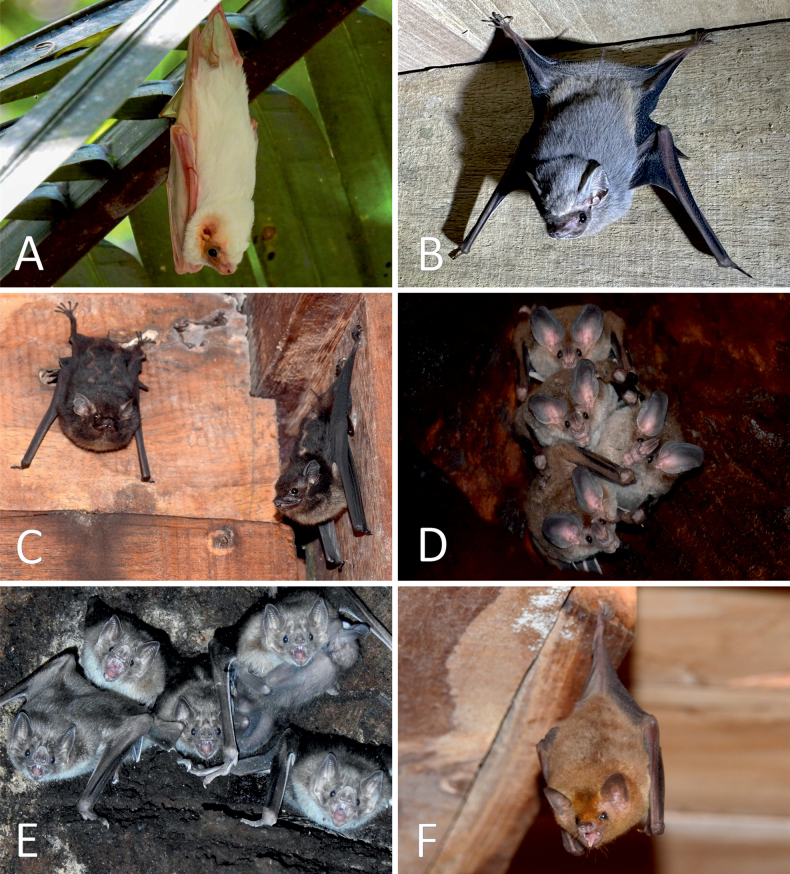
Chiropterans directly observed at the Karen Mogensen Wildlife Refuge. **A.***Diclidurus albus*; **B.***Balantiopteryx plicata*; **C.***Saccopteryx bilineata*; **D.***Chrotopterus auritus*; **E.***Desmodus rotundus*; **F.***Glossophaga soricina*. Photographs by M. Dal Zotto (**C, E, F**), F. Ciccarello (**D**), F. Minati (**B**) and C. Venegas-Elizondo (**A**).

**Figure 5.**
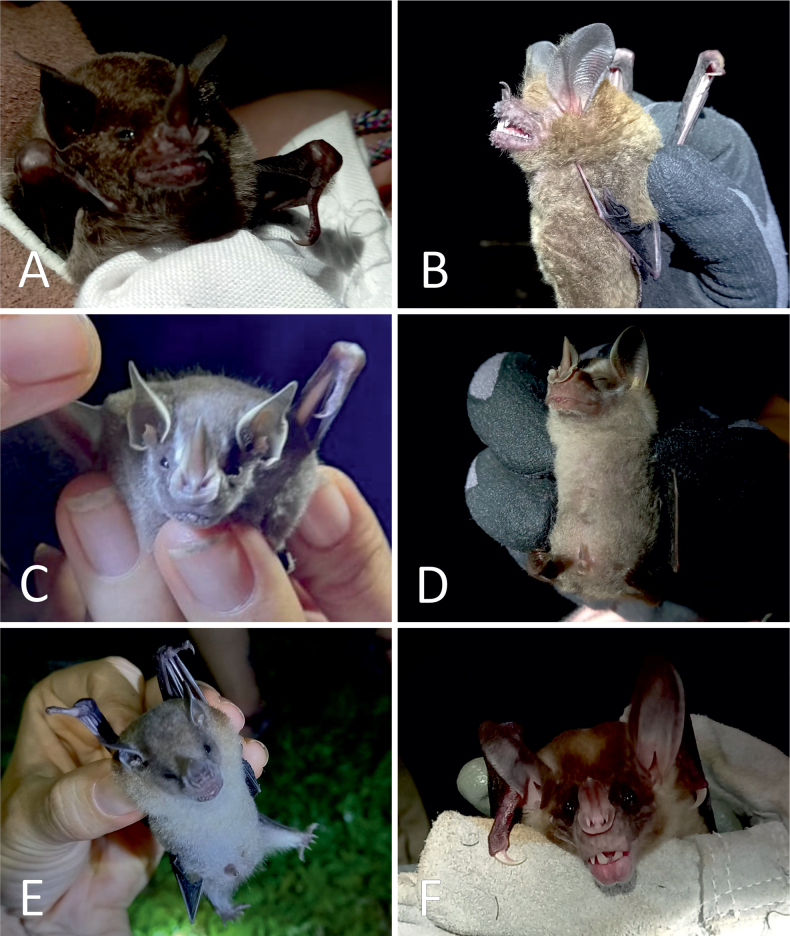
Chiropterans captured through mist netting at the Karen Mogensen Wildlife Refuge. **A.***Carollia perspicillata*; **B.***Trachops cirrhosus*; **C.***Dermanura phaeotis*; **D.***Platyrrhinus helleri*; **E.***Sturnira parvidens*; **F.***Vampyrum spectrum*. Photographs by C. Venegas-Elizondo.

Overall, data collection based on different methods allowed us to detect the presence of 60 species in 50 genera, 23 families, and 8 orders. Of these, 30 are non-volant species (29 genera, 18 families, and 7 orders; Figs 2, 3), and 30 are chiropterans (21 genera, 5 families; Table 1; Figs 4, 5). Camera trapping led from January to April 2019 revealed the presence of 13 species of mammals belonging to 11 families and six different orders (Table 1).

**Table 1.** Checklist of the mammals of the Karen Mogensen Wildlife Refuge. The systematisation, the scientific and common names follow the Mammal Diversity Database (2025). Abbreviations. Sampling methods, CT: camera trapping; DO: direct observation; MI: mist netting; TS: track/scat finding; VO: listening of vocalisation. *, species detected through camera trapping from January to April 2019.

Taxon	Vernacular name	Sampling method
** DIDELPHIMORPHIA **		
** Didelphidae **		
*Caluromys derbianus*	Derby’s woolly opossum	DO
Didelphis marsupialis*	Common opossum	CT, DO, TS
*Philander opossum*	Gray four-eyed opossum	DO
*Marmosa zeledoni*	Zeledon’s mouse opossum	DO
** PILOSA **		
** Myrmecophagidae **		
Tamandua mexicana*	Northern tamandua	CT, DO, TS
** CINGULATA **		
** Dasypodidae **		
*Dasypus novemcinctus*	Nine-banded armadillo	DO, TS
** ARTIODACTYLA **		
** Cervidae **		
Odocoileus virginianus*	White-tailed deer	CT, DO, TS
** Tayassuidae **		
Dicotyles tajacu*	Southern collared peccary	CT, DO, TS
** PRIMATES **		
** Cebidae **		
Cebus imitator*	Panamanian white-faced capuchin	CTDO, TS
** Atelidae **		
*Alouatta palliata*	Mantled howler	DO, TS, VO
** CARNIVORA **		
** Canidae **		
*Canis latrans*	Coyote	DO, TS, VO
*Urocyon cinereoargenteus*	Gray fox	DO
** Felidae **		
*Leopardus wiedii*	Margay	DO
Leopardus pardalis*	Ocelot	CT, DO, TS
*Herpailurus yagouaroundi*	Jaguarundi	CT, DO, TS
Puma concolor*	Puma	CT, TS
** Mephitidae **		
Conepatus semistriatus*	Striped hog-nosed skunk	CT, DO, TS
Spilogale angustifrons*	Southern spotted skunk	CT, DO
** Mustelidae **		
Eira barbara*	Tayra	CT, DO, TS
*Lontra annectens*	Northern neotropical river otter	CT, TS
** Procyonidae **		
Nasua narica*	White-nosed coati	CT, DO, TS
*Potos flavus*	Kinkajou	DO
*Procyon lotor*	Northern raccoon	CT, DO, TS
** RODENTIA **		
** Heteromyidae **		
*Heteromys salvini*	Salvin’s spiny pocket mouse	DO
** Cricetidae **		
*Oryzomys couesi*	Coues’s rice rat	DO
*Sigmodon hirsutus*	Southern cotton rat	DO
** Erethizontidae **		
*Coendou mexicanus*	Mexican hairy porcupine	DO
** Dasyproctidae **		
Dasyprocta punctata*	Central American agouti	CT, DO, TS
** Cuniculidae **		
Cuniculus paca*	Paca	CT, DO, TS
** Sciuridae **		
*Sciurus variegatoides*	Variegated squirrel	DO
** CHIROPTERA **		
** Emballonuridae **		
*Balantiopteryx plicata*	Gray Sac-winged bat	DO
*Diclidurus albus*	Northern ghost bat	DO, MI
*Saccopteryx bilineata*	Greater Sac-winged bat	DO, MI
** Natalidae **		
*Natalus mexicanus*	Mexican funnel-eared bat	DO
** Noctilionidae **		
*Noctilio leporinus*	Greater bulldog bat	DO
** Phyllostomidae **		
*Artibeus jamaicensis*	Jamaican fruit-eating bat	DO, MI
*Artibeus lituratus*	Gray fruit-eating bat	DO
*Carollia perspicillata*	Seba’s short-tailed bat	DO, MI
*Carollia sowelli*	Sowell’s short-tailed bat	MI
*Carollia subrufa*	Gray short-tailed bat	MI
*Chrotopterus auritus*	Big-eared woolly bat	DO
*Dermanura phaeotis*	Pygmy fruit-eating bat	MI
*Dermanura watsoni*	Thomas’s fruit-eating bat	MI
*Desmodus rotundus*	Common vampire bat	DO, MI
*Diphylla ecaudata*	Hairy-legged vampire bat	DO
*Glossophaga commissarisi*	Commissaris’s long-tongued bat	MI
*Glossophaga leachii*	Gray long-tongued bat	DO
*Glossophaga soricina*	Pallas’s long-tongued bat	DO, MI
*Phyllostomus discolor*	Pale spear-nosed bat	MI
*Phyllostomus hastatus*	Greater spear-nosed bat	DO
*Platyrrhinus helleri*	Heller’s broad-nosed bat	DO, MI
*Sturnira parvidens*	Northern yellow-shouldered bat	DO, MI
*Trachops cirrhosus*	Fringe-lipped bat	MI
*Uroderma convexum*	Mesoamerican tent-making bat	DO, MI
*Vampyriscus nymphaea*	Stripe yellow-eared bat	DO
*Vampyrum spectrum*	Spectral bat	DO, MI
** Vespertilionidae **		
*Myotis albescens*	Silver-tipped myotis	DO
*Myotis elegans*	Elegant myotis	MI
*Myotis riparius*	Riparian myotis	MI
*Rhogeessa bickhami*	Bickham’s little yellow bat	MI

Data collection over a wider timespan allowed us to detect the presence of further 17 non-volant species, and additional 16 genera, seven families, and one order. All these species were identified by direct observations and in some cases also by camera trapping (Table 1). As for the chiropterans we could detect as many as 30 species. Of these, 19 were recognised by direct observations at roosts and further 11 were identified thanks to mist netting sessions. Overall, these species belonged to 21 genera and five families. The complete list of Chiroptera is reported Table 1.

### ﻿Taxonomic accounts

In the following section we report information on all the non-volant species recorded, with that of the most relevant species of bats from a conservation perspective. The coordinates, date, and sampling method refer to the first record of each species in the investigation period.

#### ﻿Order Didelphimorpha Gill, 1872

**Family Didelphidae Gray, 1821**

**Genus *Caluromys* J. A. Allen 1900**

##### ﻿ Caluromys derbianus

Taxon classificationAnimaliaDidelphimorphaDidelphidae

(Waterhouse, 1841)

9D5DBDE2-580C-520F-A9AC-9400E2069C1D

###### Material examined.

Costa Rica • Puntarenas Province, Karen Mogensen Wildlife Refuge; 09°52'09"N, 085°03'30"W; 290 m alt.; 26.03.2021; direct observation.

###### Identification.

Medium-sized. Characterised by a grey and reddish-orange fur, pink ears, and by a half-white and half-furred tail. The body is slender, with a prehensile tail constituting 58–67% of total length (Wainwright 2007; Astúa 2015). Uncommon in the Wildlife Refuge. Recorded in different habitat types.

#### ﻿Genus *Didelphis* Linnaeus, 1758

##### ﻿ Didelphis marsupialis

Taxon classificationAnimaliaDidelphimorphaDidelphidae

Linnaeus, 1758

AA129063-AD8F-5951-B876-ED193B324574

Fig. 3A Common opossum

###### Material examined.

Costa Rica • Puntarenas Province, Karen Mogensen Wildlife Refuge; 09°52'11"N, 085°03'28"W; 298 m alt.; 05.03.2018; direct observation.

###### Identification.

Medium-sized opossum, mainly nocturnal. Identified by shaggy grey guard hairs and pale underfur. The cheeks are characterised by a dirty yellow colour and its whiskers are black. The tail is longer than the head and body, black and white, with the white portion usually longer than the black one (Wainwright 2007; Astúa 2015). Common in the study area. Recorded almost in every habitat type in all seasons. A congener species (*D. virginiana* Kerr, 1792) occurs in northwestern Costa Rica, and it is distinguished by a tail shorter than the head and body (Reid and Gόmez Zamora 2022 and literature therein).

#### ﻿Genus *Philander* Brisson, 1762

##### ﻿ Philander opossum

Taxon classificationAnimaliaDidelphimorphaDidelphidae

(Linnaeus, 1758)

7F5CC451-14B4-5E3B-9A62-5F2674595F35

###### Material examined.

Costa Rica • Puntarenas Province, Karen Mogensen Wildlife Refuge; 09°52'05"N, 085°03'32"W; 290 m alt.; 05.02.2019; direct observation.

###### Identification.

Medium-sized opossum, with nocturnal habits. Characterised by a white chin with darker upper labia, whitish belly and feet and a nearly uniform greyish brown dorsum. The ears are large and naked, rims are black, and a pale spot is present on forehead at medial base of ears. The tail is relatively small. Nose, lips, toes, and a region above chin are pink (Wainwright 2007). Recorded in various habitat types in the study area. Some authors split *P. opossum* into eight species and consider Costa Rican populations as *P. melanurus* (Voss et al. 2018; Reid and Gόmez Zamora 2022).

#### ﻿Genus *Marmosa* Gray, 1821

##### ﻿ Marmosa zeledoni

Taxon classificationAnimaliaDidelphimorphaDidelphidae

Goldman, 1911

AA78ACE7-3B05-5DB8-AAE3-D38B0C00B607

###### Material examined.

Costa Rica • Puntarenas Province, Karen Mogensen Wildlife Refuge; 09°52'00"N, 085°03'37"W; 285 m alt.; 15.04.2018; direct observation.

###### Identification.

Small-sized opossum. The dorsal fur is dark, reddish-brown, slightly paler on body sides; it turns into a greyish-brown frosted with reddish-brown in older individuals. There is no midrostral stripe. Black eyes are surrounded by blackish-brown eye-rings (Reid 2009; Rossi et al. 2010; Astúa 2015). Uncommon. Recorded in different habitat types in the Wildlife Refuge.

#### ﻿Order Pilosa Flower, 1833

**Family Myrmecophagidae Gray, 1825**

**Genus *Tamandua* Gray, 1825**

##### ﻿ Tamandua mexicana

Taxon classificationAnimaliaPilosaMyrmecophagidae

(de Saussure, 1860)

2BBCF82A-57AF-5871-B8C1-5E40CAE9F432

Fig. 3B Northern tamandua

###### Material examined.

Costa Rica • Puntarenas Province, Karen Mogensen Wildlife Refuge; 09°52'16"N, 085°03'27"W; 302 m alt.; 07.02.2018; direct observation.

###### Identification.

Medium-large sized anteater. The coat is golden-brown. The tail is long and prehensile. This species is both terrestrial and arboreal. When walking the northern tamandua folds inwards the forefeet’s claws and places its weight on knuckles (Wainwright 2007; Reid and Gόmez Zamora 2022). Recorded in various habitat types in all seasons in the Wildlife Refuge.

#### ﻿Order Cingulata Illiger, 1811

**Family Dasypodidae Gray, 1821**

**Genus *Dasypus* Linnaeus, 1758**

##### ﻿ Dasypus novemcinctus

Taxon classificationAnimaliaCingulataDasypodidae

Linnaeus, 1758

D86468E0-74CE-5AFF-B04C-980904C8A7FE

Fig. 2A Nine-banded armadillo

###### Material examined.

Costa Rica • Puntarenas Province, Karen Mogensen Wildlife Refuge; 09°52'20"N, 085°02'25"W; 295 m alt.; 10.02.2018; direct observation.

###### Identification.

Medium-sized armadillo. Characterised by an armoured body with 8 or 9 scutes. Distinguished from the other species of armadillo that occurs in Costa Rica, i.e., the northern armadillo *Cabassous centralis* Miller, 1899, by its close narrow ears, the long snout, an armoured tail and a noticeably arched carapace (Wainwright 2007; Pommer-Barbosa et al. 2023). Recorded in the Wildlife Refuge in various habitat types in all seasons. A very recent study based on both morphological and molecular data split *D. novemcinctus* into 4 distinct species. Following these results Costa Rican populations can be considered as belonging to two of these: *D. fenestratus* Peters, 1864 and *D. mexicanus* Peters, 1864 (Barthe et al. 2025).

#### ﻿Order Artiodactyla Owen, 1848

**Family Cervidae Goldfuss, 1820**

**Genus *Odocoileus* Rafinesque, 1832**

##### ﻿ Odocoileus virginianus

Taxon classificationAnimaliaArtiodactylaCervidae

(Zimmermann, 1780)

0446B1CF-1FE9-5795-8E40-0F3E0069E3E6

###### Material examined.

Costa Rica • Puntarenas Province, Karen Mogensen Wildlife Refuge; 09°52'13"N, 085°03'27"W; 296 m alt.; 18.01.2018; direct observation.

###### Identification.

Large-sized deer. The upperparts are pale to orange, the face shows conspicuous white and black markings. Long neck, head elongated, legs thin and strong. It usually carries its head high while moving. Only males have antlers, which are branched and longer than those of the other deer that occurs in Costa Rica, i.e., the brocket deer *Mazama americana* Erxleben, 1777 (Wainwright 2007). In the Wildlife Refuge this species is recorded in every habitat type in all seasons. A recent study addressed the phylogeny of all New World deers (Odocoileini) and found that *O. virginianus* should be a paraphyletic taxon (Gutiérrez et al. 2017).

#### ﻿Family Tayassuidae Palmer, 1897

**Genus *Dicotyles* Cuvier, 1816**

##### ﻿ Dicotyles tajacu

Taxon classificationAnimaliaArtiodactylaTayassuidae

(Linnaeus, 1758)

03ADAF4E-12A8-55A2-98CB-FEA8E2C6CB64

Fig. 2B Southern collared peccary

###### Material examined.

Costa Rica • Puntarenas Province, Karen Mogensen Wildlife Refuge; 09°52'16"N, 085°03'24"W; 293 m alt.; 30.01.2018; direct observation.

###### Identification.

Medium-large sized mammal, often gregarious. The coat is dark grey grizzled, with light grey or pale tan. It has an inconspicuous pale tan collar that stretches from the top of the shoulder to the back of the cheeks. The triangular-shaped head is proportionally huge, and the snout is typically pig-like. The legs are short and thin. Young individuals are characterised by a reddish-brown fur (Wainwright 2007). Common in the Wildlife Refuge. Recorded in every habitat type in all seasons.

#### ﻿Order Primates Linnaeus, 1758

**Family Cebidae Bonaparte, 1831**

**Genus *Cebus* Erxleben, 1777**

##### ﻿ Cebus imitator

Taxon classificationAnimaliaPrimatesCebidae

(Thomas, 1903)

138FBBEE-E203-558E-89E7-225A7954D3D1

Fig. 2C Central American white-faced capuchin

###### Material examined.

Costa Rica • Puntarenas Province, Karen Mogensen Wildlife Refuge; 09°51'55"N, 085°03'40"W; 279 m alt.; 20.01.2018; direct observation.

###### Identification.

Medium-sized primate, typically characterised by a black fur, except for chest, shoulders, and frontal part of the head. The face is pink coloured. Tail elongated and prehensile. It cannot be confused with other primates in the region. This species was formerly included in *C. capucinus* (Linnaeus, 1758), whose range is currently limited to South America (Boubli et al. 2012). Even though common and widespread in the Wildlife Refuge and in many parts of Costa Rica this species results globally vulnerable (Beal et al. 2020; IUCN 2025).

#### ﻿Family Atelidae Gray, 1825

**Genus *Alouatta* Lacépède, 1799**

##### ﻿ Alouatta palliata

Taxon classificationAnimaliaPrimatesAtelidae

(Gray, 1849)

FCEA2072-84B8-50A6-91A5-39DDA5B98362

Fig. 2D Mantled howler

###### Material examined.

Costa Rica • Puntarenas Province, Karen Mogensen Wildlife Refuge; 09°52'10"N, 085°03'20"W; 294 m alt.; 15.01.2018; direct observation.

###### Identification.

Medium-sized primate with dark fur and a brown to orange mantle on sides and rump, particularly marked in males. Face is black. Males show particularly visible white testicles. Frequently recorded thanks to its call, resembling a powerful roar in males (Wainwright 2007; Reid and Gόmez Zamora 2022). In line with the other monkey that occurs in the Wildlife Refuge (*C. imitator*, see above) *A. palliata* is relatively common and widespread in the study area and in several areas of Costa Rica, but is considered globally vulnerable (Beal et al. 2020; IUCN 2025).

#### ﻿Order Carnivora Bowdich, 1821

**Family Canidae von Waldheim, 1817**

**Genus *Canis* Linnaeus, 1758**

##### ﻿ Canis latrans

Taxon classificationAnimaliaCarnivoraCanidae

Say, 1823

4C0B229E-24C1-50AE-BF11-F61B22D7F15A

###### Material examined.

Costa Rica • Puntarenas Province, Karen Mogensen Wildlife Refuge; 09°52'19"N, 085°03'48"W; 384 m alt.; 11.02.2018; vocalisations.

###### Identification.

Medium-sized canid, related to the Eurasian golden jackal *Canis aureus* (Linnaeus, 1758) both for morphology and eco-ethology (Wainwright 2007). The colour of fur varies from grey to different yellow-brown tones in the upperparts and usually has paler underparts. The snout is elongated, the ears are triangular and held erect, and the tail is long with a black tip. Adapted to several habitat types thank to the extremely varied diet. Its range is rapidly expanding from North America to South America. At present the coyote is reported only along the Pacific slope of Costa Rica (Hernández-Hernández and Chávez 2021; Reid and Gόmez Zamora 2022). In the Wildlife Refuge the coyote is recorded year-round, mainly in open areas near or within dry forest habitats.

#### ﻿Genus *Urocyon* Baird, 1857

##### ﻿ Urocyon cinereoargenteus

Taxon classificationAnimaliaCarnivoraCanidae

(von Schreber, 1775)

A06B322C-BAAD-52B3-8FBB-8A4D7C7C0320

###### Material examined.

Costa Rica • Puntarenas Province, Karen Mogensen Wildlife Refuge; 09°52'28"N, 085°03'27"W; 382 m alt.; 02.02.2019; direct observation.

###### Identification.

Relatively small canid, smaller than the coyote, with rather short legs. The fur is mainly grey in the upperparts and brownish-rufous in the underparts; throat and cheeks white. The colours of the upper and lower sides are delimited by a brownish band. The ears are triangular and held erect, the tail is bushy with black tip. The throat is white and the face grey, and the sides of the neck, the abdomen and the base of the tail are reddish. This species is one of the few canids able to climb trees (Hernández-Hernández and Chávez 2021; Reid and Gόmez Zamora 2022). Occasionally recorded in dry habitats in the Wildlife Refuge.

#### ﻿Family Felidae von Waldheim, 1817

**Genus *Leopardus* Gray, 1842**

##### ﻿ Leopardus wiedii

Taxon classificationAnimaliaCarnivoraFelidae

(Schinz, 1821)

A2346353-23A5-592D-B59A-2291A7D78F02

###### Material examined.

Costa Rica • Puntarenas Province, Karen Mogensen Wildlife Refuge; 09°51'51"N, 085°03'35"W; 275 m alt.; 15.03.2021; direct observation.

###### Identification.

Small to medium-sized spotted cat, smaller than the ocelot (*L. pardalis*; see below). It may be confused with *L. pardalis*, but the tail is proportionately longer (ca. 70% of head and body length), the eyes are larger and the snout is more protruding (Wainwright 2007). Mainly arboreal, differing from the other cats recorded in the Wildlife Refuge (Beal et al. 2020). Rare; recorded in forested habitats in the study area. This species is uncommon in Costa Rica and considered Near Threatened at a global level (IUCN 2025).

##### ﻿ Leopardus pardalis

Taxon classificationAnimaliaCarnivoraFelidae

(Linnaeus, 1758)

0815F808-E1E4-536E-8E25-EBFB296CCAF9

Fig. 3F Ocelot

###### Material examined.

Costa Rica • Puntarenas Province, Karen Mogensen Wildlife Refuge; 09°52'19"N, 085°03'31"W; 334 m alt.; 15.01.2018; tracks.

###### Identification.

Medium-sized spotted cat. The fur is usually short, pale sandy brown to pale yellow. The body is entirely covered with black spots, which on the flanks become elongated rosettes with brown centres and form a striped pattern on the sides. It may be confused with *L. wiedii* (see above), but larger and with a proportionately shorter tail, approximately 45% of the head and body length (Wainwright 2007; Reid and Gόmez Zamora 2022). In the study area the ocelot is recorded in various habitat types in all seasons.

#### ﻿Genus *Herpailurus* Severtzov, 1858

##### ﻿ Herpailurus yagouaroundi

Taxon classificationAnimaliaCarnivoraFelidae

(É. Geoffroy Saint-Hilaire, 1803)

3D0DDDF8-3A60-5944-BC18-D9BFAB193DDD

###### Material examined.

Costa Rica • Puntarenas Province, Karen Mogensen Wildlife Refuge; 09°52'24"N, 085°03'30"W; 362 m alt.; 15.02.2019; camera trapping.

###### Identification.

Medium-sized cat. Elongated body, long tail and proportionately short legs. Fur varies in colour from black-grey to reddish-brown. The black phase jaguarundi can look similar to *Eira barbara* (see below) but can be distinguished by a thinner tail and the absence of a white patch on the throat (Wainwright 2007). Mainly diurnal, unlike other cats of the Wildlife Refuge, which are nocturnal or cathemeral (Beal et al. 2020; Reid and Gόmez Zamora 2022). Uncommon in the Wildlife Refuge. Recorded in various habitat types.

#### ﻿Genus *Puma* Jardine, 1834

##### ﻿ Puma concolor

Taxon classificationAnimaliaCarnivoraFelidae

(Linnaeus, 1771)

9F9C8788-1800-53D7-B122-2FD211FB694C

Fig. 3E Puma

###### Material examined.

Costa Rica • Puntarenas Province, Karen Mogensen Wildlife Refuge; 09°52'24"N, 085°03'30"W; 362 m alt.; 10.04.2018; camera trapping.

###### Identification.

Large-sized cat. The only larger felid in Costa Rica is the jaguar *Panthera onca* (Linnaeus, 1758) which was present in the Wildlife Refuge until the 1970s (L. A. Mena Aguilar pers. obs.). The fur varies in colour from pale to reddish brown. Often black and white facial markings and a black tip on the tail. It may be confused with the red phase of *H. yagouaroundi* but is considerably larger (Wainwright 2007). The most widespread wildcat of the world, with a range expanding from Alaska to the southern tip of South America (Reid and Gόmez Zamora 2022). Records of females with cubs confirm that this species breeds within the Wildlife Refuge or in its vicinity (see also the Discussion chapter). Rare in the study area; recorded in different habitat types.

#### ﻿Family Mephitidae Bonaparte, 1845

**Genus *Conepatus* Gray, 1837**

##### ﻿ Conepatus semistriatus

Taxon classificationAnimaliaCarnivoraMephitidae

(Boddaert, 1785)

1C7F78C3-770D-5211-B86B-A83366D342E4

###### Material examined.

Costa Rica • Puntarenas Province, Karen Mogensen Wildlife Refuge; 09°52'11"N, 085°03'28"W; 295 m alt.; 21.01.2018; direct observation.

###### Identification.

Medium-sized. The fur of the trunk is black with two distinctive white stripes that run from the forehead to the rump. The white stripes along the body size distinguish this skunk species from the southern spotted skunk (*Spilogale angustifrons*; see below). The tail is bushy, black at the base and then white. The snout is rather large, pig-like (Wainwright 2007; Reid and Gόmez Zamora 2022). In the Wildlife Refuge this species is recorded in various habitat types in all seasons.

#### ﻿Genus *Spilogale* Gray, 1865

##### ﻿ Spilogale angustifrons

Taxon classificationAnimaliaCarnivoraMephitidae

Howell, 1902

8F7E72FB-B46F-536D-8179-D1E02775AFDA

###### Material examined.

Costa Rica • Puntarenas Province, Karen Mogensen Wildlife Refuge; 09°52'09"N, 085°03'25"W; 306 m alt.; 31.03.2019; camera trapping.

###### Identification.

Small-sized skunk, with short legs and elongated body. The fur is black, with white stripes and spots. The tail is proportionately short, mainly white, black at the base. Occasionally recorded in the study area in transitional forest. In Costa Rica it is known only from the northwestern part of the country (Reid and Gόmez Zamora 2022). Further remarks on the range of *S. angustifrons* are reported in the Discussion.

#### ﻿Family Mustelidae von Waldheim, 1817

**Genus *Eira* Hamilton Smith, 1842**

##### ﻿ Eira barbara

Taxon classificationAnimaliaCarnivoraMustelidae

(Linnaeus, 1758)

C0813C96-CCD6-5A96-9F46-8AEC974BBEB1

Fig. 3C Tayra

###### Material examined.

Costa Rica • Puntarenas Province, Karen Mogensen Wildlife Refuge; 09°52'27"N, 085°03'28"W; 378 m alt.; 09.02.2018; direct observation.

###### Identification.

Medium-sized mustelid, with an elongated body and neck, short limbs, and a long, hairy tail. The fur is all dark except for a slightly paler head and a whitish patch on the throat. Easily distinguished from other carnivores recorded in the Wildlife Refuge (i.e., jaguarundi above and northern neotropical river otter, see below) by its dark bushy tail, and often arboreal habits (Wainwright 2007). In the Wildlife Refuge this species is recorded in various habitat types in all seasons.

#### ﻿Genus *Lontra* Gray, 1843

##### ﻿ Lontra annectens

Taxon classificationAnimaliaCarnivoraMustelidae

Major, 1897

7F063239-AC2E-5238-AF10-3059454CB54B

Fig. 3D Northern neotropical river otter

###### Material examined.

Costa Rica • Puntarenas Province, Karen Mogensen Wildlife Refuge; 09°52'11"N, 085°03'31"W; 300 m alt.; 26.02.2018; camera trapping.

###### Identification.

Medium-sized mustelid. The body is long and slim, the limbs are short, and the tail is long and stout, larger at its base. The fur is all dark brown, slightly paler around the throat and snout (Wainwright 2007; Beal et al. 2020). Central American populations of neotropical river otter were considered *L. longicaudis*, but a recent study separated them into a distinct species: the northern neotropical river otter *L. annectens* (de Ferran et al. 2024). Anyway, the neotropical river otter *L. longicaudis* is listed as Near Threatened at a global scale (IUCN 2025). Rare in the Wildlife Refuge, recorded mainly in rainy season (May–December) only along rivers and streams running through forested habitats.

#### ﻿Family Procyonidae Gray, 1825

**Genus *Nasua* Storr, 1780**

##### ﻿ Nasua narica

Taxon classificationAnimaliaCarnivoraProcyonidae

(Linnaeus, 1766)

A44F7C93-743F-5F9F-A28B-6E77B07023FF

Fig. 2E White-nosed coati

###### Material examined.

COSTA RICA • Puntarenas Province, Karen Mogensen Wildlife Refuge; 09°52'10"N, 085°03'28"W; 296 m alt.; 15.01.2018; direct observation.

###### Identification.

Medium-sized procyonid, with an elongated and rather slender body. The upperparts are chestnut to orange-brown, often paler on neck and shoulders. Throat and top of the muzzle are white. Typical white spots above or around the eyes. The muzzle is elongated with blackish snout. The tail is long, with a variable ring pattern, and is often held vertically (Wainwright 2007). Fairly common in the Wildlife Refuge. Recorded in various habitat types in all seasons. The white-nosed coati was formerly considered a single species with *N. nasua* (Linnaeus, 1766), which is exclusive of South America (Pereira et al. 2018; Pommer-Barbosa et al. 2023).

#### ﻿Genus *Potos* É. Geoffroy Saint-Hilaire & Cuvier, 1795

##### ﻿ Potos flavus

Taxon classificationAnimaliaCarnivoraProcyonidae

(von Schreber, 1774)

7529F1CE-7343-536F-9FDE-46343D78AAB1

###### Material examined.

Costa Rica • Puntarenas Province, Karen Mogensen Wildlife Refuge; 09°52'25"N, 085°03'30"W; 370 m alt.; 20.04.2018; direct observation.

###### Identification.

Medium-sized. The fur is golden yellow and rather woolly. The head is broad, with a short snout and small and rounded ears. The tail is long. Normally nocturnal and arboreal. The kinkajou is the only New World carnivore with a prehensile tail (Reid and Gόmez Zamora 2022). Recorded in the study area in different habitat types.

#### ﻿Genus *Procyon* Storr, 1780

##### ﻿ Procyon lotor

Taxon classificationAnimaliaCarnivoraProcyonidae

(Linnaeus, 1758)

72476C12-6C25-5D36-B85E-83366C1BC969

###### Material examined.

Costa Rica • Puntarenas Province, Karen Mogensen Wildlife Refuge; 09°52'16"N, 085°03'22"W; 308 m alt.; 25.01.2018; camera trapping.

###### Identification.

Medium-sized procyonid with a robust body and short legs. The face has a typical black mask, covering the eyes and cheeks and extending from the snout to the forehead and across the middle of the eyes. Another very similar species of *Procyon*, *P. cancrivorus* (Cuvier, 1798), is present in Costa Rica; this second species is mainly linked only to streams and beaches, and its range is limited to the central and southern Pacific slope of the country (Wainwright 2007; Beal et al. 2020; Reid and Gόmez Zamora 2022). In the Wildlife Refuge the northern raccoon is recorded in every habitat type in all seasons.

#### ﻿Order Rodentia Bowdich, 1821

**Family Heteromyidae Gray, 1868**

**Genus *Heteromys* Desmarest, 1817**

##### ﻿ Heteromys salvini

Taxon classificationAnimaliaRodentiaHeteromyidae

Thomas, 1893

04907BA6-2131-59A4-9092-27AFAD2E0B61

###### Material examined.

Costa Rica • Puntarenas Province, Karen Mogensen Wildlife Refuge; 09°52'04"N, 085°03'33"W; 286 m alt.; 13.02.2019; direct observation.

###### Identification.

Small-sized. The upperparts are grey-brown to dark grey, while the underside, legs and feet are totally white or cream coloured. The tail length is typically equal to the head and body length, making this spiny pocket mouse unique compared to the three congeneric species found in Costa Rica, whose distributions do not overlap with its range (Reid and Gόmez Zamora 2022). Occasionally recorded in the study area in moist and transitional forests.

#### ﻿Family Cricetidae Fischer, 1817

**Genus *Oryzomys* Baird, 1857**

##### ﻿ Oryzomys couesi

Taxon classificationAnimaliaRodentiaCricetidae

(Alston, 1877)

73371B04-4C13-509A-894D-4B406420138F

###### Material examined.

Costa Rica • Puntarenas Province, Karen Mogensen Wildlife Refuge; 09°52'10"N, 085°03'28"W; 297 m alt.; 13.02.2021; direct observation.

###### Identification.

Small-sized rodent. The upperparts are brown to orange-brown with some blackish hair. The underside is pale, whitish. The ears are relatively small and the tail is rather long and bicolor. The feet are white dorsally (Reid and Gόmez Zamora 2022). Recorded on a few occasions in clearings within forested areas or close to buildings in the study area.

#### ﻿Genus *Sigmodon* Say & Ord, 1825

##### ﻿ Sigmodon hirsutus

Taxon classificationAnimaliaRodentiaCricetidae

(Burmeister, 1854)

B8FFA551-D7BD-5364-929F-1C5F3CDDF168

###### Material examined.

Costa Rica • Puntarenas Province, Karen Mogensen Wildlife Refuge; 09°52'12"N, 085°03'27"W; 298 m alt.; 20.01.2023; direct observation.

###### Identification.

Small-sized rodent, with a grey-brown fur, paler below. The skin of the hind feet is rather dark. The nose is blunt, the ears are round and hairy. The tail is shorter than the head and body length (Reid and Gόmez Zamora 2022). Recorded on a few occasions in clearings within forested areas or close to buildings in the Wildlife Refuge.

#### ﻿Family Sciuridae de Waldheim, 1817

**Genus *Sciurus* Linnaeus, 1758**

##### ﻿ Sciurus variegatoides

Taxon classificationAnimaliaRodentiaSciuridae

Ogilby, 1839

57EE38A8-D41F-5EC3-992E-710AEDF500E5

###### Material examined.

Costa Rica • Puntarenas Province, Karen Mogensen Wildlife Refuge; 09°52'09"N, 085°03'28"W; 296 m alt.; 19.01.2018; direct observation.

###### Identification.

Small to medium sized rodent, with a long furry tail. The hair is extremely variable. Due to this characteristic at least seven subspecies are recognised in Costa Rica. All except *E. v. melania* – living in the southwestern part of the country – show a dark tail frosted with evident hairs, normally white to cream. The subspecies found in the Karen Mogensen Wildlife Refuge is *S. v. atrirufus*, characterised by a totally orange fur, except for a black midline on its back. This subspecies is endemic to southern Nicoya Peninsula (Mammal Diversity Database 2025). Some authors consider this species as belonging to genus *Echinosciurus* (de Abreu-Jr et al. 2020; Reid and Gόmez Zamora 2022). Fairly common in the Wildlife Refuge. Recorded in various habitat types in all seasons.

#### ﻿Family Erethizontidae Bonaparte, 1845

**Genus *Coendu* Lacépède, 1799**

##### ﻿ Coendou mexicanus

Taxon classificationAnimaliaRodentiaErethizontidae

(Kerr, 1792)

CCCFDD29-1BF3-562A-BA54-8E1ED147A177

###### Material examined.

Costa Rica • Puntarenas Province, Karen Mogensen Wildlife Refuge; 09°52'33"N, 085°03'12"W; 397 m alt.; 20.04.2019; direct observation.

###### Identification.

Medium-sized rodent with a black body and a pale-yellow head. Most of the hairs conceal yellowish spines, which are shorter and scarce underside. The head and ears are small, the nose is large and pink. The tail – thicker at the base – is prehensile, a unique feature among the Costa Rican rodents (Hernández-Hernández and Chávez 2021; Reid and Gόmez Zamora 2022). Rare in the investigated area. Recorded in transitional forest habitats.

#### ﻿Family Dasyproctidae Bonaparte, 1838

**Genus *Dasyprocta* Illiger, 1811**

##### ﻿ Dasyprocta punctata

Taxon classificationAnimaliaRodentiaDasyproctidae

Gray, 1842

A9496FFC-DD24-5025-A6F5-090E4E1FB6E6

Fig. 2F Central American Agouti

###### Material examined.

Costa Rica • Puntarenas Province, Karen Mogensen Wildlife Refuge; 09°52'12"N, 085°03'28"W; 397 m alt.; 18.01.2018; direct observation.

###### Identification.

Medium-sized rodent, with a slim and elongated body. The ears are short, the tail is barely visible, and the hind feet are relatively longer than the forefeet. The fur varies in colour from reddish-brown to yellowish-brown. It can be distinguished from *Cuniculus paca* (see below) – another medium-to large-sized rodent widespread in Costa Rica – mainly by the lack of white spots and smaller size (Wainwright 2007). Fairly common in the Wildlife Refuge. Recorded in every habitat type in all seasons.

#### ﻿Family Cuniculidae Miller & Gidley, 1918

**Genus *Cuniculus* Brisson, 1762**

##### ﻿ Cuniculus paca

Taxon classificationAnimaliaRodentiaCuniculidae

(Linnaeus, 1766)

C23D17F1-DA5F-532C-903B-FA3C9DB1438D

###### Material examined.

Costa Rica • Puntarenas Province, Karen Mogensen Wildlife Refuge; 09°52'11"N, 085°03'29"W; 294 m alt.; 20.01.2018; direct observation.

###### Identification.

The paca is the largest rodent of Central America, reaching a head and body length of more than 700 mm (Pérez 1992), and a body mass of 6–10 kg. The ears and tail are short, the fur is reddish-brown with rounded, whitish spots in longitudinal lines on the sides of the body. It is similar to *Dasyprocta punctata* but can be distinguished by its larger size and the presence of white spotting (Wainwright 2007). Relatively common in the study area mostly in moist forested habitats.

#### ﻿Order Chiroptera Blumenbach, 1779

**Family Emballonuridae Gervais, 1855**

**Genus *Diclidurus* Wied-Neuwied, 1819**

##### ﻿ Diclidurus albus

Taxon classificationAnimaliaChiropteraEmballonuridae

Wied-Neuwied, 1820

18354E17-7C25-537D-A580-ED842E649432

Fig. 4A Northern ghost bat

###### Material examined.

Costa Rica • Puntarenas Province, Karen Mogensen Wildlife Refuge; 09°52'16"N, 085°03'26"W; 302 m alt.; 18.01.2018; direct observation.

###### Identification.

Medium-sized bat with a totally white fur. The face and ears are yellowish and the wing membrane is pink. It is characterized by a sac around tail but no wing sacs. Roosts alone or with maximum 3–4 individuals together, frequently under palm leaves. Not abundant, but regularly recorded in various forested habitats. During our study it was frequently found under the Research Station’s roof. Its stable presence in the Wildlife Refuge is of particular interest as this bat is uncommon throughout Costa Rica (Wainwright 2007; Reid and Gόmez Zamora 2022).

#### ﻿Family Phyllostomidae Gray, 1825

**Genus *Vampyrum* Rafinesque, 1815**

##### ﻿ Vampyrum spectrum

Taxon classificationAnimaliaChiropteraPhyllostomidae

(Linnaeus, 1758)

2EBC3A2F-C559-5BD9-BEE5-76173AC4C92A

Fig. 5F Spectral bat

###### Material examined.

Costa Rica • Puntarenas Province, Karen Mogensen Wildlife Refuge; 09°52'14"N, 085°03'27"W; 296 m alt.; 10.12.2021; mist netting.

###### Identification.

Very large bat, the biggest in the New World. The upperparts are dark brown to orange-brown, with a pale white line from shoulders to rump; the fur ventrally is greyish. Nose leaf is whitish and cup-shaped. The ears are large and rounded. The tail is absent and the feet and claws are robust and elongated. Normally roosts in family groups in hollow trees. The spectral bat is a predator that feeds on a wide variety of vertebrates, including marsupials, rodents, and several birds. Rare; recorded on a few occasions in the study area. This is one of the most relevant mammals of the Wildlife Refuge from a conservation perspective, as it is considered near threatened worldwide. A female with enlarged nipples was captured during a mist netting session, testifying to the successful reproduction of this species within the Wildlife Refuge or its surrounding areas (Reid and Gόmez Zamora 2022; IUCN 2025).

### ﻿Camera trapping survey

**Figure 6.**
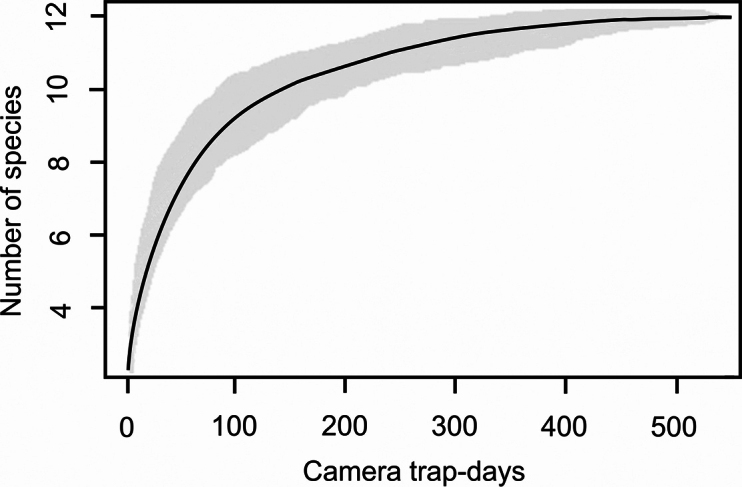
Randomised species accumulation curve (in black) and 95% confidence intervals (in grey), with the number of species detected by camera trapping from January to April 2019 in the Karen Mogensen Wildlife Refuge and sampling effort.

During the camera trapping survey from mid-January to mid-April 2019, we recorded a total of 1692 videos of 13 species (see above), 475 of which were independent detections, during the entire survey period of 102 days, corresponding to 537 trap-days. The accumulation curve of detected species showed that we reached a plateau and almost all species were captured within the first 200 trap-days (Fig. 6).

The species most frequently recorded during camera trap survey were the southern collared peccary, the white-nosed coati, the Central American agouti, and the white-tailed deer (min: 49 – max: 200 independent detections; Table 2), while the number of detections of the other species were fewer than 15. Among others, we would like to draw attention to the record of a female puma, closely followed by her two cubs.

**Table 2.** Total number of detections for each species and Mean Jacobs Index (D) for each of the defined time bands of the diel cycle. The results of Watson’s test (U^2^) of the homogeneity of diel activity patterns calculated through Kernel Density function are provided; *, p-value < 0.001. Classification of species active mostly during the day, mostly at night or cathemeral, and trophic level.

Species	Detections		D				U^2^	Activity cycle	Diet
	Total	Independent	Sunrise	Day	Sunset	Night			
Southern collared peccary	1003	200	-0.42	0.21	0.42	-0.45	1.64*	Mostly diurnal	Herbivore
White-nosed coati	345	80	-0.42	0.84	-0.45	-0.94	1.91*	Diurnal	Omnivore
Central American agouti	154	80	-0.05	0.57	0.40	-1.00	1.41*	Mostly diurnal	Herbivore
White-tailed deer	102	49	-0.37	0.38	0.23	-0.48	0.36*	Mostly diurnal	Herbivore
Common opossum	20	15	–	–	–	–	–	Nocturnal	Omnivore
Ocelot	23	14	–	–	–	–	–	Nocturnal	Carnivore
Northern tamandua	13	11	–	–	–	–	–	Mostly nocturnal	Carnivore^1^
Paca	11	11	–	–	–	–	–	Nocturnal	Herbivore
Puma	11	7	–	–	–	–	–	Cathemeral	Carnivore
Tayra	5	4	–	–	–	–	–	Diurnal	Omnivore
Striped hog-nosed skunk	3	3	–	–	–	–	–	Nocturnal	Omnivore
Panamanian white-faced capuchin	2	1	–	–	–	–	–	Diurnal	Omnivore

^1^ Although an insectivore, the northern tamandua is classified as a carnivore in the PanTHERIA database (Jones et al. 2016).

### ﻿Activity patterns and tourist impact

During the sampling period from mid-January to mid-April 2019, tourists were present at the Karen Mogensen Wildlife Refuge on 52 days and absent on 50 days. The total number of independent detections was 234 on days with tourists and 241 on days without tourists (Table 3). Four species were the most abundant and they were detected more than 40 times: southern collared peccary, white-nosed coati, Central American agouti, and the white-tailed deer. In total, their detections in relation to the presence and absence of tourists were 209 and 200, respectively (Table 3). Six species have a diurnal activity cycle, five species are nocturnal, and only the puma has a cathemeral behaviour (Table 2; Botts et al. 2020). Carnivorous species and omnivorous species were three and five, respectively, and their activity patterns were calculated together due to the low number of carnivores detections.

**Table 3.** Number of detections divided into days with tourists and days without tourists in the Karen Mogensen Wildlife Refuge. Coefficients of overlap (∆; mean and 95% confidence intervals) and Watson’s two-sample test (Two-sample U^2^) between the activity patterns of species and species groups.

	Number of detections		∆ [95% CI]	Two-sample U^2^
	Tourists present	Tourists absent		
**Species**				
Southern collared peccary	111	89	0.86 [0.77–0.92]	0.06
White-nosed coati	37	43	0.82 [0.68–0.92]	0.08
Central American agouti	39	41	0.79 [0.65–0.90]	0.06
White-tailed deer	22	27	0.72 [0.56–0.86]	0.05
**Group**				
Whole community	234	241	0.90 [0.84–0.94]	0.07
Diurnal species	210	204	0.89 [0.82–0.93]	0.08
Nocturnal species	27	27	0.78 [0.62–0.91]	0.07
Herbivores	177	163	0.88 [0.81–0.93]	0.05
Omnivores and carnivores	64	71	0.78 [0.67–0.88]	0.07

**Figure 7.**
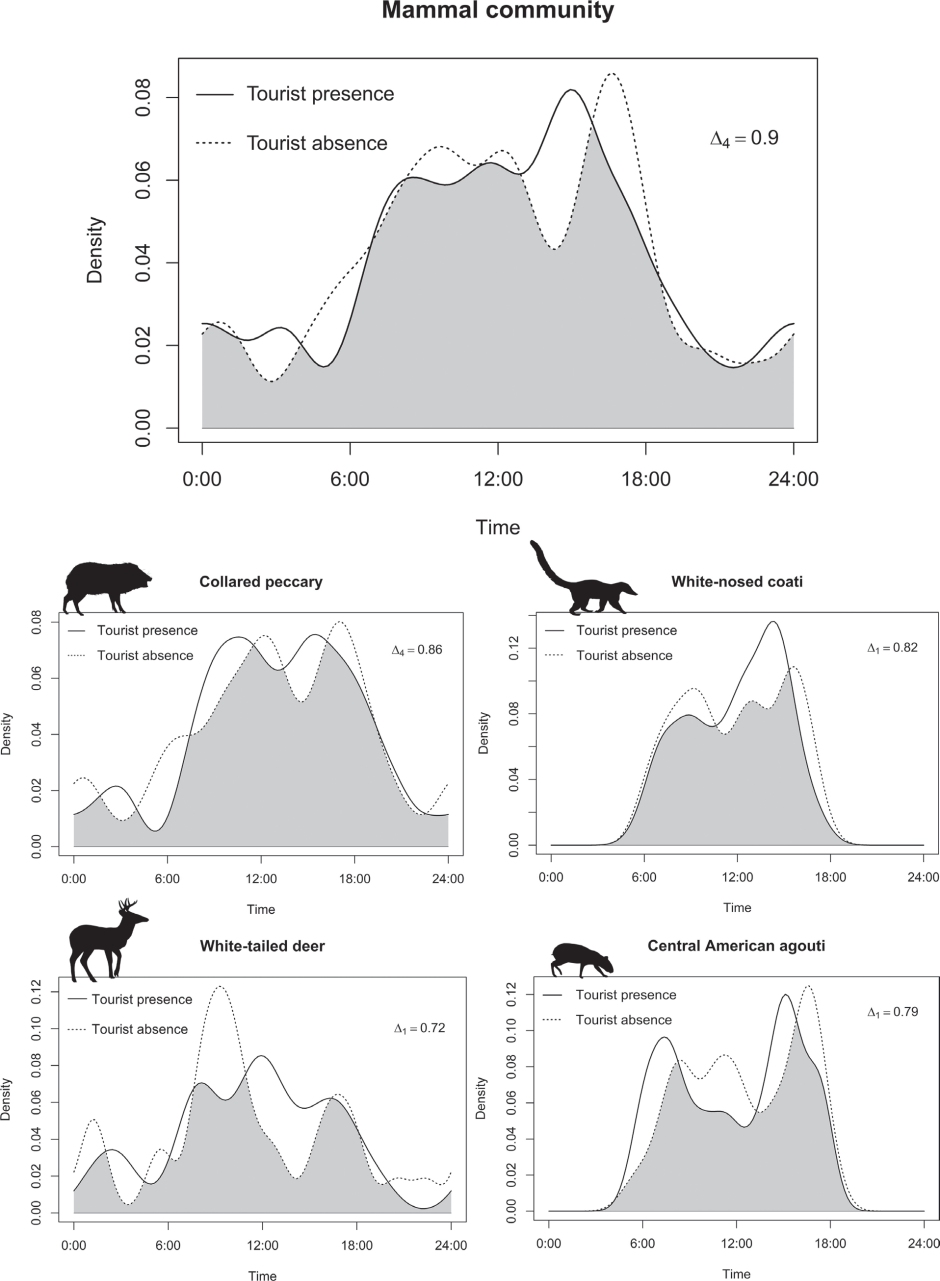
Overlap in daily activity patterns of the whole mammal community and of the most abundant species in days with tourists and in those without tourists at the Karen Mogensen Wildlife Refuge from January to April 2019. Silhouette icons from PhyloPic.org: collared peccary by Steven Traver, white-nosed coati by Margot Michaud, white-tailed deer by Andy Wilson and Central American agouti by Graham Montgomery.

**Figure 8.**
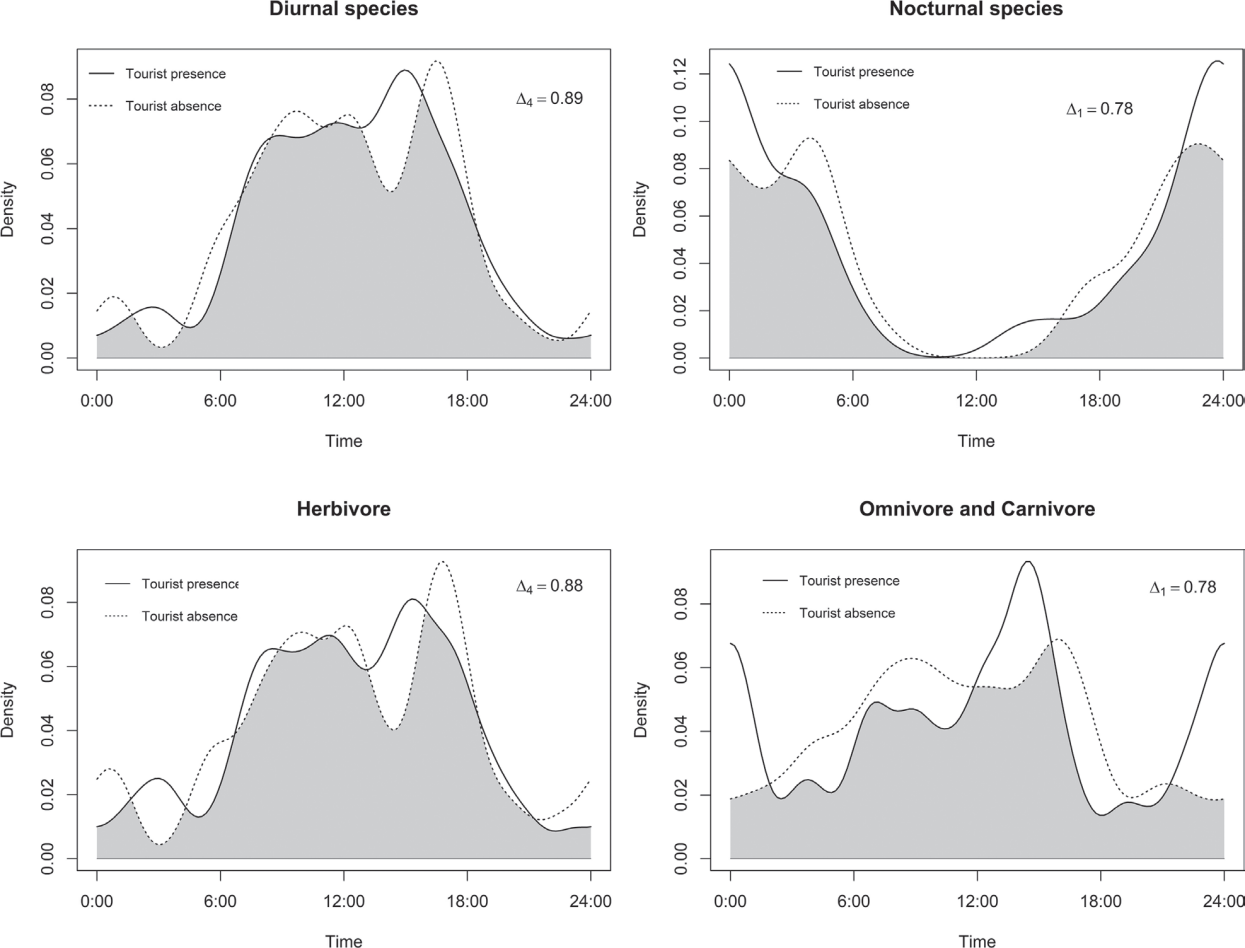
Overlap in daily activity patterns of the mammals of the Karen Mogensen Wildlife Refuge in days with tourists and in those without tourists from January to April 2019. Species grouped according to their activity cycle (nocturnal, diurnal) and diet (herbivores, omnivores, and carnivores).

For all species and groups of species based on the functional traits (activity cycle and diet), the presence or absence of tourists in the protected area did not cause any significant change in the diel activity patterns (Figs 7, 8). Indeed, the Watson’s two-sample tests performed between each comparison were not significant and the overlap coefficients were always greater than 0.72, indicating a high overlap (Table 3; Figs 7, 8).

## ﻿Discussion

Overall, our research represents the first comprehensive investigation of the mammals of the Wildlife Refuge, revealing a high species richness that represents more than 25% of all mammal species documented for Costa Rica. We highlight that the species richness of non-volant mammals of the Wildlife Refuge is substantially in line with other neotropical areas of recent investigation (e.g., Da Silva et al. 2018; Hoskins et al. 2018; Pommer-Barbosa et al. 2023), or even higher if we consider that most of these studies covered larger areas. Furthermore, our findings suggest that the current tourism management in the Karen Mogensen Wildlife Refuge seems to be effective, as we did not detect significant shifts in species’ diel activity between days with and without tourist presence. This may indicate that, currently, human disturbance associated with tourism does not appear to substantially affect the short-term temporal activity of mammal species.

Undoubtedly, the mammal community of the area consists of various small-sized terrestrial species, but during the investigation only three species belonging to two families (one Heteromyidae and two Cricetidae) have been occasionally recorded and identified. Dedicated investigations are needed for this component of the local mammalian diversity. On the contrary, regarding the chiropterans, although the results may be rather provisional, the record of 30 species indicates a high species richness, especially in relation to the small extension of the Wildlife Refuge. This number corresponds approximately to ca 25% of the Costa Rican species of bats known to date, with representatives of five out of nine families present in the country as a whole (York et al. 2019).

Some significant considerations regarding conservation issues can be drawn by our records. Two of the species reported, the mantled howler and the Panamanian white-faced capuchin, are globally Vulnerable and decreasing population trend (IUCN 2025). As for these primates, we highlight that the populations inhabiting the Wildlife Refuge show a good conservation status with more groups inhabiting the forested environment. Furthermore, one non-volant recorded species, i.e., margay, is globally Near Threatened and with decreasing populations. Besides, the status of the recently recognised northern neotropical river otter *L. annectens* is still Not Evaluated, even though until 2024 this species was considered *L. longicaudis*, whose status is Near Threatened (IUCN 2025). In addition, other three species are not threated but are characterised by globally decreasing populations, namely jaguarundi, kinkajou, and Derby’s woolly opossum (IUCN 2025). Another interesting information is the record of southern spotted skunk, as this species was not known throughout most of Costa Rica, and its southernmost global range borders correspond to the inner part of northern Costa Rica (IUCN 2025; GBIF 2025). Thus, our data extend the global range of this Mephitidae to northwestern Costa Rica, specifically to southern Nicoya Peninsula.

Further remarks concern the chiropterans. The spectral bat *Vampyrum spectrum* (Fig. 5) is rare in Costa Rica, globally Near Threatened and with decreasing populations (IUCN 2025). A nursing female was recorded in December 2022, highlighting that this species is not a sporadic occurrence in the area (authors pers. obs.). In addition, the northern ghost bat *Diclidurus albus* (Fig. 4) is an uncommon species throughout the whole country (Wainwright 2007; Loza et al. 2018; Morazán-Fernández et al. 2023). This bat was observed regularly over the years, but with no more than 3–4 individuals. The chiropterans detected include frugivorous (e.g., *Artibeus* spp., *Dermanura* spp.), insectivorous (e.g., *Myotis* spp.), carnivorous (e.g., *V. spectrum*), and haematophagous (the vampire bats *Desmodus rotundus* and *Diphylla ecaudata*) taxa. The high diversity along with the variety of feeding habits of these animals, known to be bioindicators (Wainwright 2007 and literature therein), emphasise the remarkable level of ecosystem preservation of the Wildlife Refuge.

The camera trapping led for a 4-month period allowed us to register 13 species in 537 trap-days. Taking into consideration the short period and the presence of four almost strictly arboreal species among the 30 non-volant included into the final checklist, we underscore the usefulness of this data collection method for stablishing baseline information on the mammal community of an area.

A number of studies based on or at least including this kind of data collection have been conducted in several forested neotropical locations in recent years, focusing on medium and large-sized mammals. Specifically for Costa Rica, a recent study by Beal et al. (2020) revealed the presence of 19 species for a 2-year period (1440 trap-nights) in one of the most diverse areas of the country: Piedras Blancas National Park. Similarly, Botts et al. (2020) recorded 24 species during a 10-year period (59,919 trap-days) and at 12 different sites in several locations of the country. As for other neotropical countries, for instance Hoskins et al. (2018) recorded 24 species in the Cusuco National Park, Honduras, based on different methods and during a 10-year study period (ca 900 trap-days). Da Silva et al. (2018) reported 26 species in the Iguaçu National Park, Brazil, based on camera traps at 37 sites and for an 8-month period in two different years (over 9,000 trap-days). An investigation of patches of semi-deciduous Atlantic Forest in Minas Gerais, Brazil, revealed the presence of 16 species of mammals; the study was carried out with 16 camera traps for a 2-month period (more than 900 trap-days; Costa et al. 2019). Another study in a Brazilian area, the Cazumbá-Iracema Reserve, revealed the presence of 15 species by means of 5 camera traps utilised for one year (240 trap-days; Oliveira and Calouro 2020). Jansen et al. (2020) reported 24 species in an area within the Chiquitano Dry Forest, Bolivia, using 26 camera-traps for an 8-month period (1,700 trap-days). A total of 20 species was detected in more than 2,000 ha of forest remnants in Conceição dos Ouros, Brazil (6,120 trap-days; Vilas Boas et al. 2022). Generally, as reported above, among recent studies carried out in different forested neotropical contexts, the species richness revealed is higher on average (sometimes more than twice) than the diversity that came to light in our camera trapping survey. Nevertheless, it must be noted that all these studies covered broader areas and/or longer timespans, and most of them used a higher number of camera traps, which meant a higher number of trap-days (often at least twice the trap-days in our study). If we only consider research in areas analogous to the Karen Mogensen Wildlife Refuge in terms of both size and habitat typology, and based on the use of a similar number of camera traps, the species richness recorded is largely comparable to the diversity revealed by the camera trapping activity of the present study (e.g., Oliveira and Calouro 2020: 15 species vs 13 species recorded in the Karen Mogensen Wildlife Refuge). A general consideration related to our camera trapping survey is that, as our data collection was derived from the use of camera traps placed 40–50 cm immediately above ground level, information regarding mammals with mostly arboreal activity is sparse (i.e., Panamanian white-faced capuchin) or totally lacking, as in the case of kinkajou, Derby’s woolly opossum, mantled howler, and Mexican hairy porcupine. Despite this, it must be noted that two partially arboreal species (northern tamandua and white-nosed coati) were included in the analyses because of the relatively high number of records.

The camera trapping survey allowed to gather information for studying the impact of tourism on the local mammal community. Most literature studies demonstrated that tourist activities alter the wildlife activity patterns. In a global review, Gaynor et al. (2018) revealed a strong effect of humans on daily patterns, increasing wildlife nocturnality. This shift from natural patterns has implications on fitness population persistence, community interactions, and evolution but may facilitate human-wildlife coexistence (Gaynor et al. 2018). Oberosler et al. (2017) in a protected area in the Italian Alps, found direct impact of humans on the mammals’ activity pattern: all species significantly decreased their activity during the hours most frequented by tourists. Matthews et al. (2006) and Belotti et al. (2012) demonstrated that tourist activities altered the activity patterns of black bear (*Ursus americanus*) and Eurasian lynx (*Lynx lynx*), respectively. Díaz-Ruiz et al. (2016) argued that human disturbance is a factor that affected the activity pattern of red fox (*Vulpes vulpes*), known to exhibit a behavioural plasticity, decreasing its diurnality in areas with high human presence. Conversely, in the Lapa Rios Ecolodge Natural Reserve in Costa Rica, a recent study highlighted no indication that visitation and tourism activity have negative impacts on wildlife (Lopez Gutierrez et al. 2020). The authors, comparing the hours of activity between tourists and wildlife, declared that species patterns seem to be driven by specific ecological requirement and behaviour of animals rather that by human activity. The study area and the management strategies described by Lopez Gutierrez et al. (2020) are different from the studies discussed above, but very similar to those of the Karen Mogensen Wildlife Refuge. In particular, both areas present human activities related to ecotourism in the broadest sense (Fletcher 2012; Hunt et al. 2015; Lopez Gutierrez et al. 2020). A small part of the Karen Mogensen Wildlife Refuge is crossed by a single narrow road that is not accessible to standard motor vehicles. Furthermore, the number of tourists is limited, and they are always accompanied by guides. Acknowledging that some species are under-represented in the overall pool of observations, our results show, in agreement with Lopez Gutierrez et al. (2020), that diurnal activity patterns of mammals and species groups that share similar traits, i.e., the activity cycle and the diet, do not appear to be affected by human presence. However, our study focused specifically on diel activity patterns to explore potential short-term behavioural responses of mammals to tourism. While this approach offers valuable insight into temporal shifts in activity between day with and without tourists, it represents only one aspect of species’ ecological responses. Therefore, we cannot exclude the possibility that tourism may influence other aspects of mammal ecology, such as spatial distribution and habitat use. Further research with broader methodological approaches and larger sample sizes, with larger temporal scale, would be necessary to comprehensively evaluate the potential effects of tourism on wildlife. Nevertheless, our current findings did not reveal significant short-term behavioural changes associated with tourist presence, and the Wildlife Refuge supports a high level of mammalian diversity, suggesting that current tourism levels may not be exerting strong negative impacts. More generally, the information gained highlights the importance of the investigated area for conservation. Despite its relatively limited extension, it represents an area of high biodiversity and an essential part of the Biological Corridor of the Nicoya Peninsula. A further relevant reason for the present study stems from the increased knowledge of the mammal community in one of the least studied regions of Costa Rica, though characterised by a globally high conservation interesting environment, i.e., the threatened tropical dry forest ecosystem of Mesoamerica (Janzen 1988; Huettmann 2015).

Our investigation underscores that the wilderness of the Wildlife Refuge is undergoing a period of renaissance since its creation in 1996, marked by the presence of a rich animal diversity with a diversified vegetation, similarly to other well-known and more popular parks and natural reserves in Costa Rica, all of which have greater extensions. As reported by a former investigation on the avifauna, several parts of the area have shown a clear regeneration process, with the growth of secondary forest surrounding the patches of primary forest already present before the birth of the Wildlife Refuge (Dal Zotto et al. 2017). The species richness reported here proportionately matches recent ornithological studies conducted in the area, which proved the presence of more than 220 bird species (Dal Zotto et al. 2017; Lopez et al. 2023; authors pers. obs.). Undeniably, there is clear evidence that the area is recovering from historical anthropogenic disturbances, as the mammal community shows signs of ecological regeneration.

The present study allowed us to define and understand the current dynamics occurring between and among the mammal species of the Wildlife Refuge. This information is fundamental for future conservation plans, in order to permit a positive coexistence between the local human communities and the autochthonous fauna. Wildlife conservation can be effective only if innovative strategies at a local scale take into account the coupled nature of social and ecological systems (Guerrero and Wilson 2016). This may seem easy, but, for instance, we must know that the jaguar (*Panthera onca*), once living in the area, disappeared during the 1970s precisely because they were killed by local farmers who responded to the predation of their cattle by this felid. Our investigation showed that the puma, which had also disappeared from the area for several years, is now back in the Wildlife Refuge. In fact, the puma was detected in 2018, after years without any indications of its presence (L. A. Mena Aguilar pers. obs.) and our study confirmed its first documented reproduction within the area, thanks to some videos portraying females with two cubs. Its permanent presence must be taken into consideration in order to avoid repeating the fate suffered by the jaguar. We must also consider that this last felid is recolonising the north-west of Costa Rica, on the border with Nicaragua, so we cannot exclude that, in the next few years, it will come to populate again the area that today corresponds to the Refuge. More generally, in Costa Rica, areas such as the targeted one, although protected by national laws as Wildlife Refuges, need more custody from poaching, which unfortunately has become increasingly frequent in recent years (authors pers. obs.), as well as from the presence of free-ranging dogs, which have been shown to have deleterious effects on wildlife, acting as predators or competitors to native species, especially in fragmented habitats or well-preserved areas of tropical environments (Carvalho et al. 2019). These issues are not easy to solve since they require long-term projects to help the local people understand the importance of the fauna and flora that surround them, both in terms of ecosystemic concerns and of economic returns. It is our hope that, with the additional knowledge gained from surveys such as this one, local governors and stakeholders will make prudent decisions to preserve and defend these areas and allow the development or the continuation of conservation projects. To this end, a positive scenario results from the recent conversion of the private Karen Mogensen Reserve into the Wildlife Refuge (Refugio de Vida Silvestre), which is included in the network of the public protected areas of Costa Rica.

## Supplementary Material

XML Treatment for Caluromys derbianus

XML Treatment for Didelphis marsupialis

XML Treatment for Philander opossum

XML Treatment for Marmosa zeledoni

XML Treatment for Tamandua mexicana

XML Treatment for Dasypus novemcinctus

XML Treatment for Odocoileus virginianus

XML Treatment for Dicotyles tajacu

XML Treatment for Cebus imitator

XML Treatment for Alouatta palliata

XML Treatment for Canis latrans

XML Treatment for Urocyon cinereoargenteus

XML Treatment for Leopardus wiedii

XML Treatment for Leopardus pardalis

XML Treatment for Herpailurus yagouaroundi

XML Treatment for Puma concolor

XML Treatment for Conepatus semistriatus

XML Treatment for Spilogale angustifrons

XML Treatment for Eira barbara

XML Treatment for Lontra annectens

XML Treatment for Nasua narica

XML Treatment for Potos flavus

XML Treatment for Procyon lotor

XML Treatment for Heteromys salvini

XML Treatment for Oryzomys couesi

XML Treatment for Sigmodon hirsutus

XML Treatment for Sciurus variegatoides

XML Treatment for Coendou mexicanus

XML Treatment for Dasyprocta punctata

XML Treatment for Cuniculus paca

XML Treatment for Diclidurus albus

XML Treatment for Vampyrum spectrum

## ﻿Additional information

### Use of AI

No use of AI was reported.

### Author ORCIDs

Matteo Dal Zotto https://orcid.org/0000-0003-1386-7626

### Data availability

All of the data that support the findings of this study are available in the main text.
